# Psychoeducation

**DOI:** 10.4103/0019-5545.55082

**Published:** 2009

**Authors:** G. Swaminath

**Affiliations:** Department of Psychiatry, Dr. B R Ambedkar Medical College, Kadugondanahalli, Bangalore - 560 045, Karnataka, India

## A CHANGING PROFILE

The ‘new age’ clients' propensity to quiz the doctor on various aspects of diagnosis and management is certainly novel. At the least it causes discomfort to the physician who had long become used to the patient's blind, unflinching confidence and trust, along with a reluctance to question the doctor on aspects which bothered him/her. The discomfiture becomes more acute when patients and their relatives confront one with information which they have gleaned through alternative sources, such as helpful ‘informed others’, their own reading and the electronic media, not the least among which is surfing the internet. The professionals are inclined to treat these condescendingly, and tend to be on their guard in the inquirers' presence and resent the extra time taken by them. This stems from a paternalistic belief that physicians have the best interest of the patient in mind, and misinterpret the queries of the patient and relatives as being a sign of distrust.

This is not necessarily true. While there seems to be a creeping distrust of the medical profession among patients nowadays, this change is in considerable measure because today's patients and care givers want information, education and a complete understanding of the condition and its treatment[1] and they believe it to be their fundamental right. The younger the patient, the more demanding he or she tends to be.[1] Over time, the autonomy of the patient has justifiably taken precedence over beneficence and non malfeasance.[2] Many writers on contemporary bioethics believe that all intentional suppression of pertinent information violates a patient's autonomy rights and violates the fundamental duties of a physician.[2]

## THE GOOD DOCTOR

In a 1988 National Research Corporation (NRC) survey, physicians were rated by consumers as ‘high quality’ by their willingness to maintain patient health through educational information and willingness to talk about the individual's illness and treatment.[3] In another NRC survey, consumers listed willingness to explain things as the most important criterion in selecting a physician, with other factors such as reasonable fees, telephone access, friendly office staff, convenient appointments, and a convenient office location rated much lower.[3]

Communication about how the health care system works as well as the process and prognosis of the patient's disease, is often more important in ensuring quality care than medical jargon, fancy gadgets and high tech tests to which the physicians and health care system are sometimes, wittingly or otherwise, wedded.[1] And if this comes from the health care provider, it becomes most effective and credible.[1]

Patient education (or Psychoeducation as in our case) is critical to compliance and outcome. Here patients and their relatives, by means of preliminary briefing concerning the illness, are supposed to develop a fundamental understanding of the therapy and further be convinced to commit to more long-term involvement.[4] Psychoeducation looks to combine the factor of empowerment of the affected with scientifically founded treatment expertise in as efficient a manner as possible.[4] It satisfies patient (and family) expectation, improves confidence in the physician,[1] ensures that patients get their information mainly from the health care provider[1] and less from other sources which may impart inaccurate information, as well as helps patients develop insight into their illness and therefore make better decisions and become more compliant.[1] Psychoeducation, in addition, promotes relapse prevention,[4] assists support of healthy components[4] and also strengthens the role of relatives as ‘co-therapists’ to assist the therapeutic team in their goal.[4] Thus there is an optimal combination of professional therapeutic methods and empowerment.

The elements of Psychoeducation include a triad of therapeutic interaction, clarification, and enhancing coping competence.[4] Therapeutic interaction involves establishing a relationship level wherein medical information is shared, with simultaneous respect and esteem for the subjective individual opinions of the afflicted.[4] Clarification involves professional simplification of complex facts about illness through an interactive style of providing information, induction of insight into illness and its requisite treatment measures, and a transmission of understanding as well as experiences of “enlightenment” through a two-way conveyance of information.[4] Finally there can be ‘enhancement of coping competence’ by focusing on resources and not on deficits, optimized crisis management behavior, modification of life plan, transformation of patients into “experts” of their illness (knowledge is power), and strengthening the protective potential of the family.[4]

To take an example: In schizophrenia sufferers and/or their care- givers, an attempt to clarify the term “schizophrenia” should be made, briefing them regarding positive and negative symptoms, as well as their origin (e.g. dopamine excess with disturbance in information processing), medication and side effects discussed, psychosocial measures instituted and counseling carried out on early warning signs. In addition, we ought to recommend a strategy for any crisis/relapse prevention.

Many factors influence the flow of information from the physician to the patient, These include patient anxiety, physician use of overly technical language, patient confusion, and patient misunderstanding.[5] Even after physicians believe they have “educated” their patients, the patients have acquired only about 50% of the relevant information.[6] Fewer patients follow the recommendations by physicians such as medication adherence, appointment keeping, and life style alternations (e.g. changes in diet, exercise, or unhealthy habits such as smoking). The more acute and life-threatening the illness and the less amount of change required by the recommendation, the more likely the patient will follow the advice.[5]

## HOWS AND WHYS

Strategies to improve the likelihood of the patient following advice range from education, to motivational strategies to, finally, coercion.[7] Coercion is the ultimate type of influence and should be rarely used by physician. Motivational strategies to achieve adaptive health behavior represent a higher level activity compared to education. Many argue that the physician should not be in the business of motivating the patients to change.[5] However, education is the simplest, straightforward and non controversial strategy to improve compliance.[5]

There are six steps in the process of educating a patient about his illness:[5]

- Eliciting the patient's ideas of etiology where, with an understanding of the patient's particular ideas and concerns about their illness, the physician will be better able to relieve the patient's anxieties and misconceptions and respond to their concern.[5] For example, in anxiety and panic disorder patients, reassuring them (occasionally proactively) that their physical symptoms such as headache, throat pain or chest pain or palpitations do not suggest life threatening illnesses such as cancer or heart disease, ameliorates considerable apprehension, improves the doctor-patient relationship and improves clinical outcomes.
- Providing a basic diagnosis where information should be given briefly and succinctly, as anxiety could impair comprehension.[5]
- Responding to the patient's feelings about the diagnosis where the patient is assisted to verbalize and express his emotions and the physician provides realistic hope along with emotional support, both verbal and non verbal.[5]
- Checking the patient's knowledge of the illness and explain only what he does not know, as well as correct misinformation and deficiencies in his knowledge.
- Providing details of the diagnosis and elaborate finer points in easily understandable language, using short sentences to make points clearly.[5]
- Finally, checking the patient's understanding of the problem which is a very important education skill, and ending with a query whether the patient has further questions in a manner assuming that he has questions.[5]

While educating the patient and families it should be borne in mind that the detailed knowledge of the illness, in particular regarding chances of recovery, therapy possibilities and the disease process can make the patient and/or family member stressed.[4] Therefore, one should evaluate the risks considering the psychological condition of the patient, on how much the patient already understands, and how much knowledge the patient can take up and process in their current condition.[4]

Psychoeducation does more than just meet patients' needs and expectations as they do insist on information and education.[1] Patient education enhances the perception of the physician's quality, improves patient compliance, reduces malpractice risk, and increases productivity by reducing after-hours or next-day telephone calls or emergency consultations.[1] It helps patients make informed choices. Thus is laid the foundation for achieving optimal collaboration between empowerment of the patient and professional expertise.
